# Analysis of small-sample clinical genomics studies using multi-parameter shrinkage: application to high-throughput RNA interference screening

**DOI:** 10.1186/1755-8794-6-S2-S1

**Published:** 2013-05-07

**Authors:** Mark A van de Wiel, Renée X de Menezes, Ellen Siebring-van Olst, Victor W van Beusechem

**Affiliations:** 1 Department of Epidemiology and Biostatistics, VU University Medical Center, PO Box 7057, 1007 MB Amsterdam, the Netherlands; 2 RNA Interference Functional Oncogenomics Laboratory (RIFOL), Department of Medical Oncology, VU University Medical Center, Amsterdam, the Netherlands; 3 Department of Pulmonary Diseases, VU University Medical Center, Amsterdam, the Netherlands

## Abstract

High-throughput (HT) RNA interference (RNAi) screens are increasingly used for reverse genetics and drug discovery. These experiments are laborious and costly, hence sample sizes are often very small. Powerful statistical techniques to detect siRNAs that potentially enhance treatment are currently lacking, because they do not optimally use the amount of data in the other dimension, the feature dimension.

We introduce ShrinkHT, a Bayesian method for shrinking multiple parameters in a statistical model, where 'shrinkage' refers to borrowing information across features. ShrinkHT is very flexible in fitting the effect size distribution for the main parameter of interest, thereby accommodating skewness that naturally occurs when siRNAs are compared with controls. In addition, it naturally down-weights the impact of nuisance parameters (e.g. assay-specific effects) when these tend to have little effects across siRNAs. We show that these properties lead to better ROC-curves than with the popular limma software. Moreover, in a 3 + 3 treatment vs control experiment with 'assay' as an additional nuisance factor, ShrinkHT is able to detect three (out of 960) significant siRNAs with stronger enhancement effects than the positive control. These were not detected by limma. In the context of gene-targeted (conjugate) treatment, these are interesting candidates for further research.

## Introduction

Many clinical genomics studies suffer from low power and low reproducibility caused by small sample sizes. Small sample sizes may be due to high costs per sample, low availability of genomic material (e.g. for rare diseases) or even juridical restrictions (e.g. when administering an experimental drug to patients). The philosophy behind our method is to increase power and reproducibility by retrieving as much information as possible from the vertical data direction (feature space: genes, tags, small interference RNAs (siRNAs), etc.) for estimating differential treatment effects from the horizontal data direction (sample space).

In statistical terms the latter is referred to as 'shrinkage'. In a classical setting, a shrinkage estimator is a weighted average between the estimator from the concerning feature and a pooled estimator from all features. Shrinkage of dispersion-related parameters, like *σ*^2 ^for the Normal distribution, is now commonly applied to genomics data and has been implemented in popular analysis software like limma [1]. We take a step further. We show that shrinking additional parameters, including the main parameter of interest, e.g. the treatment effect, may further enhance power and reproducibility. Our approach is an Empirical Bayes framework around a Full Bayes fitting method called Integrated Nested Laplace Approximation (INLA [2]). Here, INLA provides a fast, flexible, versatile and accurate alternative to MCMC, whereas our framework uses the high-dimensional aspect of the data to estimate priors, which effectuates shrinkage.

For the analysis of RNA-seq count data, we introduced ShrinkSeq [3]. We showed its improved performance in terms of Receiver Operating Characteristic (ROC)-curves with respect to other methods like edgeR, baySeq and DESeq, in particular when the data contain many zeros and sample sizes are small. Here, we provide several extensions and new insights: 1) ability to handle high-dimensional Gaussian data; 2) model selection properties when nuisance parameters are involved; 3) flexible and powerful inference with potentially asymmetric priors.

High-throughput (HT) RNA interference (RNAi) screens [4] are increasingly used for reverse genetics and drug discovery. Statistical methods used for HT screens data analysis are commonly borrowed from small-molecule screens or even other types of high-dimensional data methods. However, HT screens data differs from other high-dimensional data in fundamental ways. Firstly, HT screens data are more susceptible to technical effects, as the cell culture plates are handled several times over a multiple day period, and the various experimental steps are performed by a variety of equipment. Secondly, studies currently involve a very small (1-3) number of replicates per condition. Hence, statistical inference is often absent in HT RNAi studies: only fold changes and standard deviations are mentioned. Thirdly, HT screens data typically involve a large amount of observations for controls, which serve as references for condition effect but are not of primary interest, and a very limited number of observations per siRNA, which are the primary interest. This highly unbalanced design essentially means that classic statistical methods are underpowered to find siRNA-specific effects.

Ideally, HT screens contain two types of controls: a negative one, which in a treatment sensitization setting can be used to estimate the effect of treatment alone, and a positive one, which is a gene known to have an (additional) effect on the response, e.g. cell viability, when silenced. While comparisons to both controls are of interest and our method applies to both, we focus on the one relative to the positive control. Then the lack of power is particularly relevant: an siRNA can only be significant (in comparison with the positive control) when the differential siRNA-specific cell viability between treatment and 'no treatment' conditions exceeds that of the positive control. Most data information then arises from treatment effect for controls, with generally hundreds of measures per replicate, whilst the main goal typically is to draw conclusions about siRNA-specific treatment sensitivity, for which only a single measure is available per replicate. There is thus great need for methods that can borrow information across features (siRNAs), to improve the chance of finding relevant siRNAs.

In this work, we use data from a cisplatin sensitization HT screen. Cisplatin is a DNA damaging agent that has been used for over 30 years to treat cancer and is part of standard therapy for some cancer-types, including non-small cell lung cancer. Unfortunately, results are suboptimal and some patients react better than others. Chemo-resistance is a major problem, for which the molecular cause is largely unknown. Cisplatin sensitization screens aim at identifying genes involved with the sensitivity to cisplatin. This knowledge may render useful biomarkers for treatment response, and elucidate which pathways are involved in resistance to cisplatin. Moreover, the identified genes are potential targets for more effective treatment when cisplatin is combined with an inhibitor of the corresponding gene product.

The data were produced as follows. Cells from the established non-small cell lung cancer (NSCLC) cell line A549 were seeded in 96 well plates. Next day, these cells were transfected with siRNAs targeting a selected set of 960 genes, using a robotic set up. This renders two sets of 24 plates for which each well contains a pool of siRNA targeting one gene. The following day a low dose of cisplatin was added to the wells of one set of plates, while the other set received an equal volume of culture medium without cisplatin. Four days after cisplatin exposure, read out of viability commenced for both sets. This procedure was repeated twice. The positive control, *BIRC7*, is a known inhibitor of apoptosis-related genes and sensitises A549 cells to cisplatin. The negative control consists of a pool of siRNAs that do not target a known human mRNA. The cisplatin concentration used was relatively low: 0.45 *μ*g/ml. At that concentration, the reduction of viability by cisplatin in *BIRC7 *silenced cells, compared to cells treated with non-targeting siRNAs, is most profound. Reducing cisplatin in a clinical setting while preserving toxic effect on the tumor is likely to reduce cytotoxic side effects like kidney and auditory nerve damage.

The HT screen data was read into R and normalized by correcting for a plate or assay effect using a linear regression model on the logarithm of the data values, considering all six screens simultaneously. Subsequently, the treatment effect was estimated for positive controls only, also controlling for possible remaining assay-specific effects. The remaining siRNA observations, corrected for control-related treatment effect, were then studied using two alternative empirical-Bayes approaches. The first one involved studying treatment effect in the corrected siRNA data by using limma. This approach shrinks the effect's variance only, which leads to a modified *t*-test. The second one is ShrinkHT, which shrinks multiple parameters: effect variance, effect size and possibly also nuisance effects like the assay-specific ones for data with Gaussian errors.

## Methods

### Introduction to (Bayesian) shrinkage

**Figure 1:**
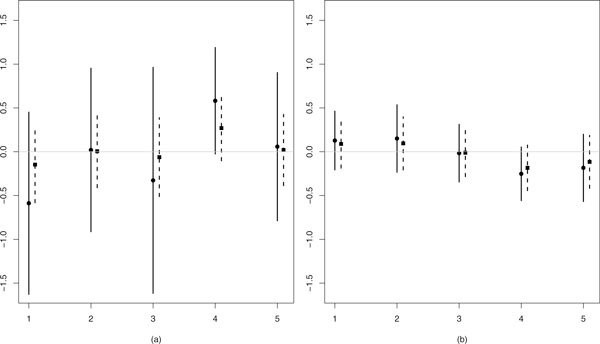
**Illustration of shrinkage effect**. Estimates of *β*_2 _(mean ± standard deviation) for five studies, using model m1 (vague prior; circle/solid line) and model m2 (shrinkage prior; square/dashed). (a): *n *= 6, (b): *n *= 40. Data are generated from model (1) with *β*_2 _= *β*_1 _= 0 and $\varepsilon_{jk}{=}^{d}N\left(0,1\right)$.

Consider a simple data set, for which features (e.g. siRNAs) *X*_2_, ..., *X_p _*are measured on *n *= 6 subjects (divided in two groups) only. For illustration purpose assume feature *X*_1 _is measured on the same 6 subjects, but also in 4 other studies (so 5 in total) with the same technology and study design. Feature *X*_1 _will be used to illustrate the potential beneficial effect of shrinkage. A simple Analysis of Variance (ANOVA)-type model for groups *j *= 1 and *j *= 2 is:

(1) $$Y_{jk}=\alpha +\beta_{j}+\varepsilon_{jk},$$

where *Y_jk _*is the data for subject *k *in group *j*, *α *is a common intercept and *ε_jk _*follows a central Normal distribution. In addition, *β*_1 _is set to zero, so *β*_2 _can be interpreted as the mean log-fold change between groups. In a Bayesian setting, model (1) is not complete: priors need to be specified for all parameters. Let us focus on the prior for *β*_2_. In a conventional Bayesian analysis, a flat prior would be used, e.g. a Gaussian with very large variance, f.e. *N*(0; 100^2^). This reflects a complete lack of prior believe or evidence on the size and direction of the log-fold change. Refer to model (1) with this prior as model m1. Now our method allows to estimate a prior from *X*_2 _..., *X_p _*(or from *X*_1 _..., *X_p_*: because *p *is large *X*_1 _has a negligible effect on the estimate, so we do not violate the 'do not use the data twice' principle). Suppose the log-fold changes tend to be much more concentrated, reflected by an estimated shrinkage prior *N*(0, τ^2^), with τ = 0.5. Use of this prior defines model m2. Suppose their is no effect for feature *X*_1_, so *β*_2 _= 0. Figure 1(a) illustrate estimates of *β*_2 _for *X*_1 _for the five studies under models m1 and m2. We observe two beneficial effects of the shrinkage prior: the means are shrunken towards zero (and hence better reproducible), and the standard deviations are markedly smaller than for the vague prior. For large sample sizes the differences between the shrunken estimates and the ones based on a vague prior diminish (see Figure 1(b)), because the data then dominates the prior.

Use of a shrinkage prior effectuates (Bayesian) shrinkage: (posterior) estimates of the feature-specific log-fold changes are shrunken towards the overall mean log-fold change by using a prior that is concentrated around this mean. Here, the high-dimensional aspect of our data plays an important role: the availability of *X*_1_, ..., *X_p_*, with *p *large, allows us to *estimate *a common prior, rather than assuming it. In our example, the shrunken prior is correctly positioned for *X*_1_. However, if the true *β*_2 _is not well supported by the prior, shrinkage may do more harm than good, because it may introduce a bias. This is the reason why we allow flexible, non-parametric priors, which can accommodate a wide variety of distributional properties such as skewness or heavy tails. Below we discuss estimation of such priors.

### Design of cisplatin sensitization HT screening experiment

The analysis of the HT screening data consists of three parts: 1) Estimation of the hyper-parameters (parameters of priors), which effectuates shrinkage; 2) Estimation of the model parameters; 3) Inference. The design of the experiment is given in Table 1. Next, we describe the model for the HT screening data.

**Table 1:** Design of the study

Measurement					
*y*_11_	*y*_21_	*y*_12_	*y*_22_	*y*_13_	*y*_23_
Untreated	Treated	Untreated	Treated	Untreated	Treated
Assay 1	Assay 1	Assay 2	Assay 2	Assay 3	Assay 3

Design of the cisplatin sensitization HT screening experiment

### Model

The model is the same for all siRNAs and hence we drop the siRNA index when not referring to a specific siRNA. Then, *Y_jk _*is the *j*^th ^observation for the *k*^th ^assay for a given siRNA, where *j *= 1 corresponds to 'no treatment' and *j *= 2 to 'treatment'.

(2) $$\begin{array}{llll} Y_{jk} & =\text{offse}{\text{t}}_{jk}+\beta_{0}+\beta_{j}^{\text{treat}}+\beta_{k}^{\text{assay}}+\varepsilon_{jk}\; & & \;\\ \varepsilon_{jk} & {=}^{d}N\left(0,\sigma^{2}\right)\; & & \;\\ \beta_{k}^{\text{assay}} & {=}^{d}N\left(0,\tau^{2}\right)for\;\;k\geq \;\;2;\;\;\beta_{\text{1}}^{\text{assay}}=0\; & & \;\\ \beta_{\text{2}}^{\text{treat}} & {=}^{d}F_{NP};\;\;\beta_{\text{1}}^{\text{treat}}=0\; & & \;\\ \sigma^{-2} & {=}^{d}\Gamma \left(\alpha_{1},\alpha_{2}\right)\; & & \;\\ & \; & \end{array}$$

Here, offset*_jk _*is trivially computed from the positive control data. It is the estimated effect of the positive controls for treatment level *j *and assay *k *using the same model as above, but with vague priors for all parameters. Since the amount of data on the positive controls is large (288 measurements), and the standard deviations of the estimates are small, we do not further propagate the uncertainty of these estimates.

The crux behind our method is that we empirically estimate hyper-parameters *τ*^2^, *α*_1_, *α*_2 _and *F_NP_*, which is a non-parametric, log-concave distribution function [5]. The latter provides maximum flexibility for the main parameter of interest, $\beta_{\text{2}}^{\text{treat}}$. In the Results section we show that this flexibility is essential for detecting significant siRNAs.

### Estimation of hyper-parameters

**Figure 2:**
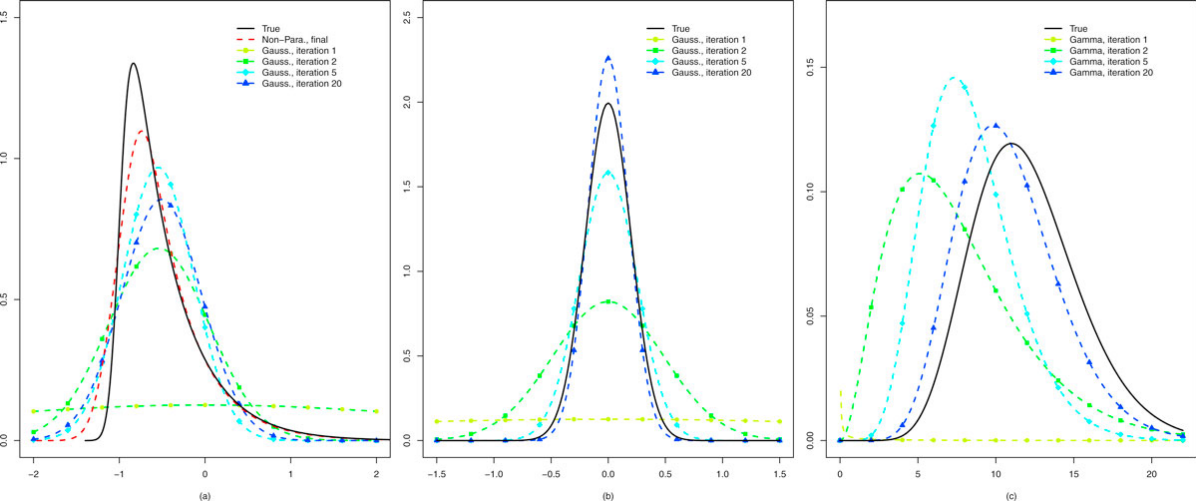
**Iterative estimates of priors**. (a) Treatment parameter $\beta_{\text{2}}^{\text{treat}}$; (b) Nuisance parameters $\beta_{k}^{\text{assay}}$; (c) Precision *σ*^-2^.

Let us first consider the empirical estimation of the Gaussian variance parameter *τ*^2^. Suppose we initially do not estimate *τ*^2 ^and just set it to some large value (eg 10^6^) which reflects a vague prior for $\beta_{k}^{\text{assay}}$. Likewise other hyper-parameters are initialized to induce vague priors, including a vague Gaussian prior to initialize *F_NP_*. Then, INLA provides posterior distributions of all parameters. Now write *m_ik _*and *v_ik _*as the posterior mean and variance of $\beta_{ik}^{\text{assay}}$, where we add index *i *to indicate siRNA *i*. In other words, *m_ik _*and *v_ik _*are means and variances conditional on the data for siRNA *i*. Using the well-known variance propagation rule, *V*(*A*) = *V_B_*(*E*(*A*|*B*)) + *E_B_*(*V*(*A*|*B*)), we may now simply re-estimate the unconditional, prior variance by

(3) $${\hat{\tau }}^{2}=\hat{V}\left(m_{ik}\right)+{\bar{v}}_{ik,}$$

where $\hat{V}$ is the sample variance estimator. Now, the procedure may be repeated until convergence, where new posteriors are computed under the updated prior variance ${\hat{\tau }}^{2}$. Figure 2(b) shows an example of central Gaussian priors that result from iterative estimates of $\tau^{2}$. The rationale behind this iterative procedure is as follows. Posteriors are a compromise between the prior and the likelihoods (the data). Hence, as long as the common prior 'conflicts' with the data, pooling the posteriors, which is what (3) does, leads to a prior that, in a global sense, is more in line with the data. Of course, here it is quintessential to have much data in the vertical direction, siRNAs, to ensure that the prior does not tune to one particular siRNA.

For non-Gaussian, parametric distributions the above moment-estimator is replaced by a maximum likelihood-based one. Importantly, the procedure above may trivially be extended to estimate multiple priors: at each iteration INLA provides posteriors given *all *priors, hence accounting for potential interdependencies, whereas the re-estimation of the priors depends only on the marginal posteriors of the concerning parameter.

Finally, let us shortly explain how *F_NP _*in model (2) is estimated. INLA can currently not deal with non-parametric priors. Hence, in the above joint iterative procedure we have to assume a parametric prior for $\beta_{\text{2}}^{\text{treat}}$, e.g. Gaussian. Denote this prior with *π*(*β*). Now suppose we want to replace this prior by a non-parametric one π*(*β*), the density corresponding to distribution function *F_NP_*. INLA returns posteriors *π*(*β|Y*) under prior *π*(*β*). The following re-weighting allows to compute a posterior π*(*β|Y*) under prior π*(*β*):

$$\pi^{*}\left(\beta |Y\right)=\frac{\pi \left(\beta |Y\right)\pi^{*}\left(\beta \right)/\pi \left(\beta \right)}{\int \pi \left(\beta |Y\right)\pi^{*}\left(\beta \right)/\pi \left(\beta \right)d\beta },$$

where the denominator scales the numerator to ensure that $\pi^{*}\left(\beta |Y\right)$ is a density integrating to one. The principle for estimating $\pi^{*}\left(\beta \right)$ (and hence *F_NP_*) is the same as with the above joint iterative procedure: iteratively and alternatingly update $\pi^{*}\left(\beta \right)$ and $\pi^{*}\left(\beta |Y\right)$. Here, the new update of $\pi^{*}\left(\beta \right)$ is just a log-concave fit to a large sample of the empirical mixture of posteriors of $\beta_{i\text{2}}^{\text{treat}}$, where log-concavity helps to stabilize the tails.

Hence, the joint iterative procedure is followed by a marginal iterative procedure (which updates only one prior) and together they provide estimates of *τ*^2^, *α*_1_, *α*_2_, *F_NP _*and the posteriors of $\beta_{i\text{2}}^{\text{treat}}$. Several (mathematical) details of the procedure, such as approximate equivalence to maximum marginal likelihood, convergence criteria and various types of parametric priors are further discussed in [3].

### Inference

An important feature of HT screening is that, due to the interpretation of the $\beta_{i\text{2}}^{\text{treat}}$ (treatment sensitization effect relative to a positive control), one is mostly interested in $\beta_{i\text{2}}^{\text{treat}}>0$ This implies that inference needs to be one-sided. This is particularly important in an FDR setting: if the inference is performed two-sided, so also aims at detecting $\beta_{i\text{2}}^{\text{treat}}<0$, this may heavily bias the results for $\beta_{i\text{2}}^{\text{treat}}>0$, even when those are a posteriori selected. The reason is that the many 'significant' results for $\beta_{i\text{2}}^{\text{treat}}<0$ (which are to be expected: many siRNAs will have smaller effects than the positive control) push the FDR downwards. This asymmetry for the positive and negative effects is further highlighted in the Results section.

In [3] we argued that in a shrinkage context the posterior probability p_0*i *_= 1 - p*_i _*= $1-P\left(\beta_{i2}^{\text{treat}}>0|Y_{i}\right)$ can be interpreted as a local false discovery rate [6], which for cut-off *t *leads to Bayesian False Discovery Rate (BFDR [7]) when averaged over all p_0*i *_≤ *t*. This can be used analogously to ordinary FDR.

## Results

### Successful estimation of the priors

To assess whether priors can be successfully estimated in this very challenging setting with only 6 measurements per siRNA, two conditions and the presence of a three-level nuisance factor (assay), we set up a simulation that is strongly motivated from the data: the model is the same as (2) except for the offset which is not relevant here. Moreover, the effect size distribution *F_NP _*is an asymmetric one: Γ(1, 0.5) shifted to the left by Δ = -1, where our main interest is in the positive tail. Finally, *α*_1 _and *α*_2 _are set to 12 and 1, respectively, such that the resulting Gamma distribution mimics the observed one for the estimates of *σ*^-2 ^and *τ *= 0.2 which implies small assay effects, as observed in the data as well. Thousand siRNAs are simulated. The results of the iterative algorithms are displayed in Figure 2.

Given the sample sizes and the number of model parameters per siRNA, the results are very accurate. From (b) and (c) we conclude that the final parametric estimates (Iteration 20) are fairly close to the true ones for the nuisance parameter and the precision. For the first, the final estimate is $\hat{\tau }=0.18$, whereas for the latter it is (${\hat{\alpha }}_{1}$, ${\hat{\alpha }}_{2}$) = (10.90, 1.01). From (a) it is clear that the non-parametric estimate can provide a substantial improvement with respect to the Gaussian one for the parameter of interest. Note that the left-tail is somewhat more difficult to estimate due to its steepness, whereas the right-tail (reflecting positive effects, in which we are interested) is very accurately estimated.

## Comparison

We compare ShrinkHT with limma in a data-based simulation set-up. Here, the functional screening data serves as a template for the sample sizes, number of features and noise levels. More specifically, we simulate two groups of three samples for 960 features from Gaussian distributions with standard deviations equalling those of the corresponding data estimates. For the main parameters of interest, the differences of means, we assume that 80% of these equal exactly 0. The remaining 20% is simulated from four differential effect size distributions: Gamma(0.5, 0.75), halfNormal(0, 0.47), Gamma(0.25, 0.75) and halfNormal(0, 0.25). Here, the half-Normal distributions contain twice the mass of the positive part of the central Normal ones. The first two distribution corresponds to a mean differential effect of δ = 0.375, whereas the latter two correspond to δ/2.

**Figure 3:**
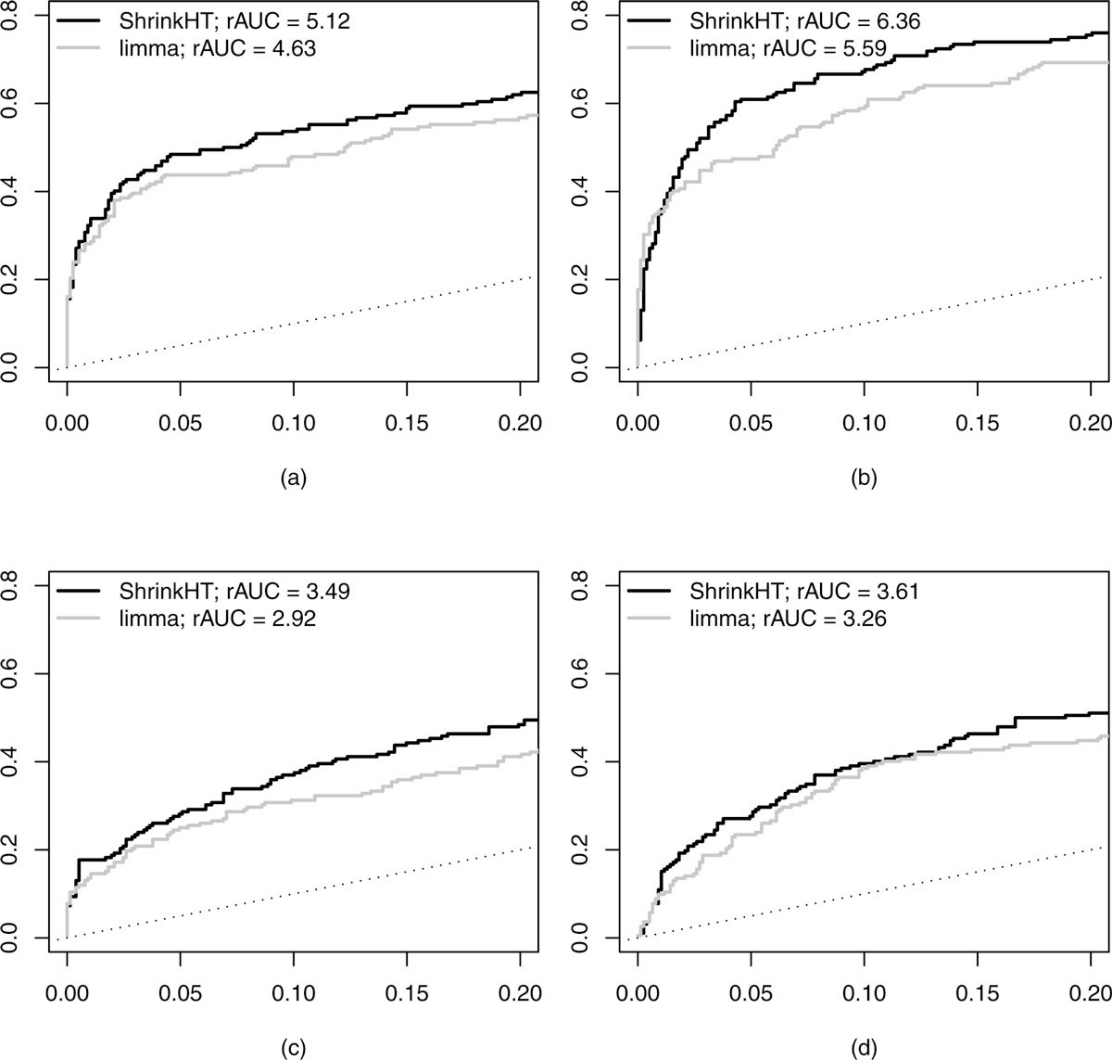
**ROC-curves for four effect size distributions**. X-axis: False Positive Rate (1-specificity), y-axis: True Positive Rate (sensitivity). (a) Gamma(0.5; 0.75); (b) halfNormal(0, 0.47); (c) Gamma(0.25, 0.75); (d) halfNormal(0, 0.25).

The simulated data were analyzed with ShrinkHT and limma. In this setting, the two approaches differ essentially in only one aspect: in addition to the standard deviations, ShrinkHT shrinks the mean differential effects whereas limma does not. After the analysis, significance results are ranked for both approaches, enabling computation of the False Positive Rate (FPR; 1-specificity) and True Positive Rate (TPR; sensitivity). Figure 3 displays the resulting ROC-curves. For all comparisons we show partial ROC-curves and partial Area-Under-the-Curve (AUC) because in a testing setting only small FPR (we use FPR ≤ 0.2) is relevant [8]. Partial AUC (pAUC) is expressed in terms of relative AUC (rAUC), rAUC = pAUC = (0.2^2 ^* 0.5), where the denominator is the expected AUC for a non-informative decision procedure.

Figure 3 clearly shows the benefit of additional shrinkage in ShrinkHT. For example, for FPR = 0.05, the TPRs of ShrinkHT are 1.11, 1.29, 1.13 and 1.16 times higher than those of limma for cases (a)-(d), respectively.

### Analysis of HT screening data

To illustrate the effect of several levels of shrinkage, we compute *eqN*: the 'equivalent number of replicates', which equals *eqN *= *N*/p^*^, with total sample size *N *= 6 and p^* ^the effective number of parameters in the model [9]. High values of *eqN *lead to more precise, and hence more reproducible, estimates of the parameters. Shrinkage can potentially decrease p^* ^and hence increase *eqN*. We consider four scenarios: 1) no shrinkage (flat priors); 2) shrinkage of sd *σ *only; 3) shrinkage of *σ *and $\beta_{\text{2}}^{\text{treat}}$; 4) shrinkage of *σ*, $\beta_{\text{2}}^{\text{treat}}$ and $\beta_{k}^{\text{assay}}$.

In scenario 1) *eqN *equals 1.5 for all siRNAs. This is to be expected, because with flat priors and Gaussian models p^* ^is just the number of free parameters [9], which is 4 in model (2). In scenario 2) *eqN *also equals 1.5 for all siRNAs, because *σ *is a hyper-parameter in the hierarchical model, which does not contribute to the computation of p^* ^[9]. Hence shrinkage of this parameter has no effect on *eqN*. For scenario 3) the mean *eqN *equals 1.73 (sd: 0.01), whereas for scenario 4) the mean *eqN *equals 3.21 (sd: 0.08). Hence, in particular shrinkage of the 2 free assay parameters has a very beneficial effect on *eqN*. In fact, under scenarios 1) and 2) sample size N should be more than doubled to obtain approximately the same value of *eqN*: *N*′ = 13 would result in eqN′ = 13 = 4 = 3.25. The gain in *eqN *for scenario 4 is due to the strong reduction of p^*^, which results from the very narrow central Gaussian prior for $\beta_{k}^{\text{assay}}$: standard deviation $\hat{\tau }=0.095$. Hence, the assay parameters are strongly shrunken towards 0, which illustrates the quasi-parameter-selection property of our method.

**Figure 4:**
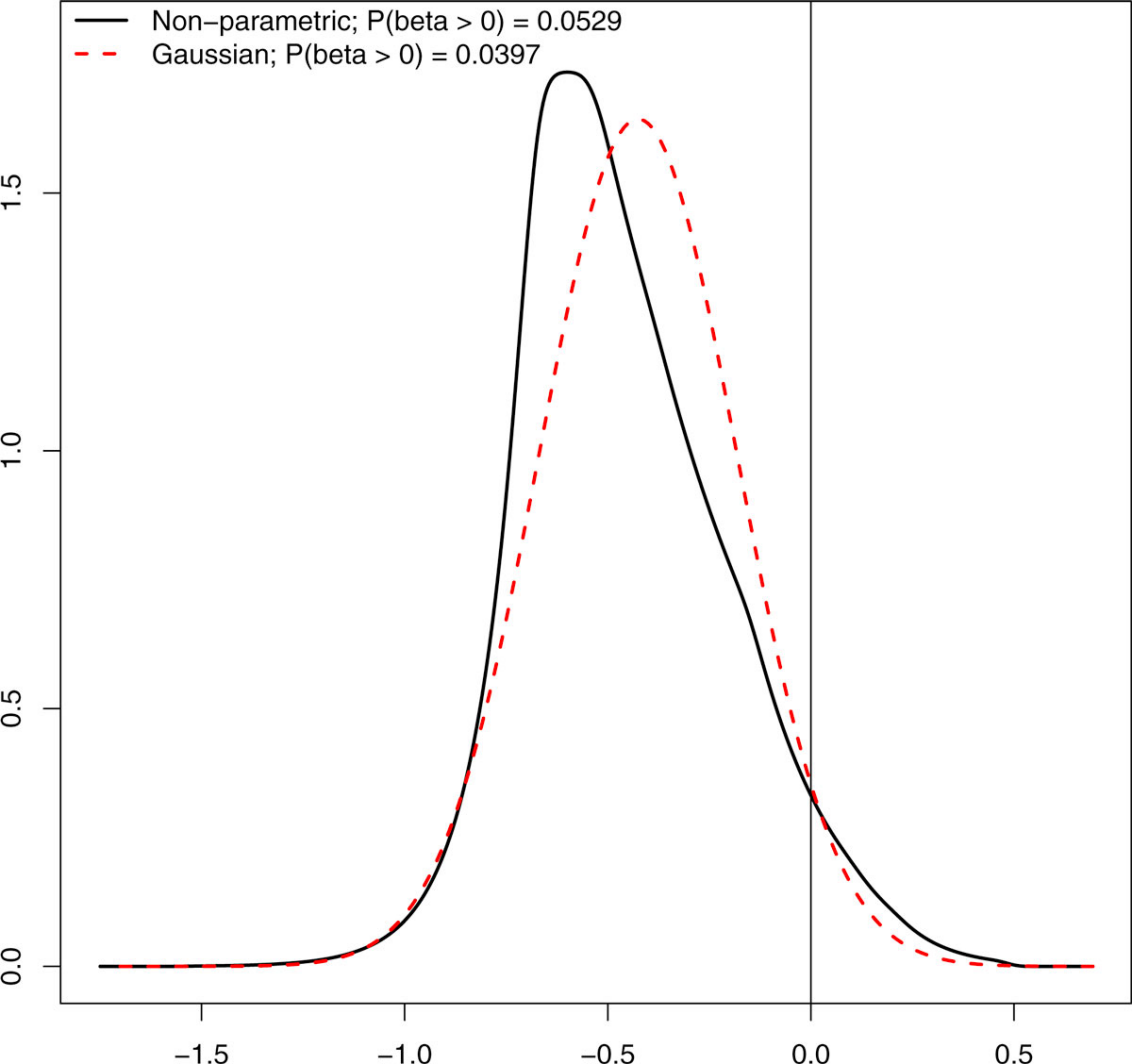
**Estimates of Gaussian and non-parametric prior**. Gaussian and non-parametric estimates of the treatment sensitization effect size $\left(\beta_{\text{2}}^{\text{treat}}\right)$ density and the prior probabilities $P\left(\beta_{\text{2}}^{\text{treat}}>0\right)$: the area under the positive part of the curves.

An interesting aspect of HT screens data is that when comparing treatment enhancement effect of siRNAs with that of the positive control it is likely that relatively many siRNAs are less effective than the positive control. Here, we show that it is quintessential to account for this potential asymmetry in the statistical analysis. Figure 4 shows the estimated Gaussian (and hence estimated) treatment effect size distribution, and the flexible, non-parametric one, which allows for skewness. Importantly, the two differ strongly on the area of primary interest, the positive area, reflected in the 0.0529/0.0397 = 1.33 times larger mass in this area for the non-parametric one. More importantly, the practical consequence is that at a 0.1 cut-off for the BFDR the non-parametric prior leads to 3 detections of siRNAs that have a stronger effect than the positive control, whereas the Gaussian prior results in only 1 detection (see Table 2). At Benjamini-Hochberg FDR cut-of 0.1, limma returns no detections when one-sided testing is used. However, the comparison with limma should be interpreted tentatively, because the true false positive rate is unknown.

**Table 2:** Results of the analysis

Id	Untreated			Treated			${\hat{\beta }}_{i\text{2}}^{\text{treat}}$	ShrinkHT NP; BFDR	ShrinkHT G; BFDR	limma FDR
608	-0.63	0.22	-0.14	-1.20	-1.29	-1.37	0.337	0.0136	0.080	0.727
749	0.22	0.28	0.43	-0.51	-0.42	-0.25	0.255	0.0362	0.115	0.413
176	0.38	0.39	0.59	-0.06	-0.31	0.11	0.175	0.0738	0.169	0.727

1^st ^column: siRNA id; 2^nd ^to 7^th ^column: log_2 _cell viability data for untreated and treated cell lines, corrected for the differential effect in positive control; 8^th ^column: estimate of treatment sensitization effect ('untreated - treated') in excess of the positive control when ShrinkHT is used with a non-parametric prior; 9^th ^and 10^th ^column: BFDRs when using a non-parametric and Gaussian prior; 11^th ^column: Benjamini-Hochberg corrected FDR for limma results

Table 2 also shows the posterior mean estimates of $\beta_{i\text{2}}^{\text{treat}}$ when using the non-parametric treatment effect size prior. So, for example, the enhancement effect of siRNA 608 on cell viability is estimated to be 2^0.337 ^= 1.26 times larger than that of the positive control. Note that our shrinkage implies that this estimate is intrinsically corrected for selection bias [10].

## Conclusion

We discussed ShrinkHT, a new method for analyzing HT screening data. Its efficiency and power were demonstrated in both simulation and real data settings. We showed that it is able to detect siRNAs that sensitize cells to cisplatin with a stronger effect than the positive control. It is important to have several candidates that exhibit strong effects, because in the trajectory of further validation many siRNAs are likely to fail. HT screens are expensive and laborious which explains the small sample sizes (often smaller than 3 vs 3) found in literature. Still, even when such small sample size studies are considered as pilots it is important to select the right features for further experiments and to have a good estimate of the false discovery rate to minimize the risk of wasting resources and time.

While we focused on the comparison with the positive control, the comparison with the negative control is at least as important. Here, the gain of ShrinkHT with respect to other methods is relatively less when considering treatment sensitization, because these effects tend to be large when compared to the negative control. On the other hand, the siRNAs that sensitize the treatment *less *than the negative control are also of interest, because these may protect the cells against the treatment. Those protective effects are likely to be smaller and less prevalent than enhancement effects. The adaptivity of the prior towards non-symmetric situations like these renders ShrinkHT very suitable to find such protective effects.

For some HT screens many siRNAs have significantly larger effects than the negative control, whereas none of these has a significantly stronger effect than the positive control (or a good positive control is lacking). For example, silencing of the commonly used positive control gene *PLK1 *usually completely kills cells irrespective of the treatment modality. Then, inference with respect to the controls is only partly helpful for selecting siRNAs that sensitize treatment for further validation. In such a case, our method allows a potentially useful alternative: inference with respect to the mean or mode. In an empirical Bayes setting, the prior can be assumed known when estimating marginal posteriors and hence also when drawing inference from these. Hence, we may base the 'null-hypothesis' on e.g. the mean of the prior of the primary parameter of interest, $\beta_{\text{treat}}$, and declare an siRNA significant if its effect is significantly larger than this mean. Since the prior mean reflects the average posterior mean over all siRNAs, such significance should be interpreted as a relative statement: the concerning siRNA has a significantly larger effect than the average. In our setting, 181 siRNAs showed such a significant effect at BFDR ≤ 0.1.

In silico validation of our approach is not straightforward. Large sample HT screens are not available, which disallows a sample splitting approach like we performed for RNA-seq data [3]. For those data we demonstrated better reproducibility of the results from our approach with respect to others. On genome-wide screens, pathway (enrichment) analysis could be useful, but our selected set of 960 genes is likely too small for this purpose. Biological validation of significant siRNAs (hits) is planned for the near future. Follow up in RNAi screens with pools of siRNAs targeting the mRNA of one gene, such as in our example data set, starts with the process called deconvolution; each of the single siRNAs from a hit pool is tested to see if it elicits the same effect. Often this is accompanied by quantification of the knock down of the targeted mRNA, in order to detect possible off target effects of the siRNAs. In this particular screen, deconvolution will be combined with a dose response curve to cisplatin to truly assess the sensitizing effect of siRNAs targeting the gene of interest.

ShrinkHT is part of the R-package 'ShrinkBayes', under the function name 'ShrinkGauss'. ShrinkBayes also includes ShrinkSeq for the analysis of RNA-seq data. The package is available from http://www.few.vu.nl/~mavdwiel. We are convinced that ShrinkHT contributes to powerful and reliable detection of siRNAs which, in the context of gene-targeted (conjugate) treatment, are interesting candidates for further validation.

## Competing interests

The authors declare that they have no competing interests.

## Authors' contributions

MvdW developed the analysis method and software (ShrinkHT). MvdW and RdM performed statistical analyses. ESvO, VvB and RdM designed the RNAi HTS experiment. ESvO performed the experiment. MvdW drafted the manuscript. All authors read and approved the final manuscript.

## Acknowledgements

We thank Ida van der Meulen-Muileman for technical assistance in liquid handling.

Ellen Siebring-van Olst is supported by the Walter Bruckerhoff Stiftung. High-throughput screens were conducted at the RNA Interference Functional Oncogenomics Laboratory (RIFOL) core facility at the VUmc Cancer Center Amsterdam, which was established with support from the Stichting VUmc CCA.

## Declarations

The publication costs for this article were shared by all authors: the Departments of Epidemiology & Bio-statisics, Medical Oncology and Pulmonary Diseases, VU University Medical Center, Amsterdam, provided funding in three equal parts.

This article has been published as part of *BMC Medical Genomics *Volume 6 Supplement 2, 2013: Selected articles from the Second Annual Translational Bioinformatics Conference (TBC 2012). The full contents of the supplement are available online at http://www.biomedcentral.com/bmcmedgenomics/supplements/6/S2.
